# Molecular characteristics and phylogenetic analysis of *Clostridium perfringens* from different regions in China, from 2013 to 2021

**DOI:** 10.3389/fmicb.2023.1195083

**Published:** 2023-07-06

**Authors:** Jia xin Zhong, Hao ran Zheng, Yuan yuan Wang, Lu lu Bai, Xiao li Du, Yuan Wu, Jin xing Lu

**Affiliations:** ^1^State Key Laboratory of Infectious Disease Prevention and Control, Chinese Center for Disease Control and Prevention, National Institute for Communicable Disease Control and Prevention, Beijing, China; ^2^Collaborative Innovation Center for Diagnosis and Treatment of Infectious Diseases, Hangzhou, China

**Keywords:** *Clostridium perfringens*, MLST (multilocus sequence typing), antimicrobial resistance (AMR), cgMLST, genomic

## Abstract

*Clostridium perfringens* (*C. perfringens*) is a significant foodborne pathogen and a common cause of intestinal diseases in both animals and humans. Our study investigated MLST, phenotypic antimicrobial resistance profiles, and resistance genes among isolates from human, animal and food. 186 *C. perfringens* isolates were obtained from nine provinces in China between 2013 and 2021. Additionally, some specific ST complexes were analyzed by cgMLST and cgSNP to investigate genetic relatedness. MLST indicated the most prevalent STs of *C. perfringens* of human and animal origin were as follows: ST221 (5/147), ST62 (4/147), ST408 (4/147), and ST493 (4/147) were predominant in humans, while ST479 (5/25) was the major type in animals. Within the same ST complex, genetically unrelated relationships or potential clustering/transmission events were further recognized by cgMLST and cgSNP, illustrating that these two methods are valuable in defining outbreaks and transmission events. All tested isolates were susceptible to vancomycin and meropenem. The rates of resistance to metronidazole, penicillin, cefoxitin, moxifloxacin, and chloramphenicol were low (metronidazole: 1.08%; penicillin: 9.68%; cefoxitin: 0.54%; moxifloxacin: 6.45%; and chloramphenicol: 3.76%). Interestingly, 49.66% of human origin were clindamycin-resistant, and 18.2% were penicillin-insensitive. Importantly, the portion of MDR isolates was significantly lower than in previous reports. The study provides an overview of the epidemiological characteristics of *C. perfringens* with different origins and hosts in China. *C. perfringens* demonstrated remarkable genetic diversity and distinct molecular features compared to antibiotic-resistance profiles from other studies.

## Introduction

*Clostridium perfringens* (*C. perfringens*) is a Gram-positive anaerobic bacterium that is abundant in nature. The bacteria can cause gas gangrene, intestinal diseases, and food poisoning in both animals and humans (Brynestad and Granum, 2002; Kiu and Hall, 2018). The significant pathogenic mechanism of *C. perfringens* is the production of a wide variety of toxins and enzymes (Li et al., 2013). *C. perfringens* is classified into seven types: A (*ɑ*), B (*ɑ*, *β*, *ƹ*), C (*ɑ*, *β*), D (*ɑ*, *ƹ*), E (*ɑ*, L), F (*ɑ*, *cpe*), and G (*ɑ*, *netB*) by a toxin-based typing scheme (Li et al., 2013).

Antibiotic-associated diarrhea (AAD) continues to be a major healthcare issue, both in hospitalized patients and in the community (Kyne et al., 2002). Many investigations have revealed that *C. perfringens* played an important role in diarrheic patients with a history of antibiotic use (Carman, 1997; Azimirad et al., 2019). According to some studies, *C. perfringens* is responsible for 5–20% of all cases of AAD and sporadic non-foodborne diarrhea (Carman, 1997; Motamedi et al., 2021). Recently, an increase in *C. perfringens* antimicrobial resistance has been noted, and some investigations have indicated that most *C. perfringens* strains of animal origin are multidrug resistant (MDR) (Xu et al., 2021; Bendary et al., 2022). However, in China the research on *C. perfringens* antimicrobial resistance is limited.

The technique of MLST is widely applied for the identification of human, animal, and foodborne pathogens, and it has also been used to analyze bacterial population diversity and investigate *C. perfringens* outbreaks (Lacey et al., 2016). With the increasing application of whole-genome sequencing (WGS), high-resolution molecular subtyping approaches such as core genome MLST (cgMLST) based on WGS have gained popularity in epidemic investigation and bacterial tracing (Abdel-Glil et al., 2021a,b). Previous research has shown that *C. perfringens* from different sources had a large amount of genetic variation (Park et al., 2016; Álvarez-Pérez et al., 2017; Lacey et al., 2018), and human strains demonstrated more variation than animal strains (Nakano et al., 2017). However, the molecular characteristicistics of *C. perfringens* isolates from different sample origins and regions in China are limited. Therefore, this research aimed to provide valuable epidemiological data on genotype distribution, antibiotic resistance, *toxinotype*, genetic diversity, and phylogenetic characteristics of *C. perfringens* from distinct geographical regions in China. The findings can provide a deeper understanding of the molecular epidemiology of *C. perfringens* in China and offer a basis for the development of rapid detection and surveillance networks.

## Materials and methods

### Sample collection

**Figure 1:**
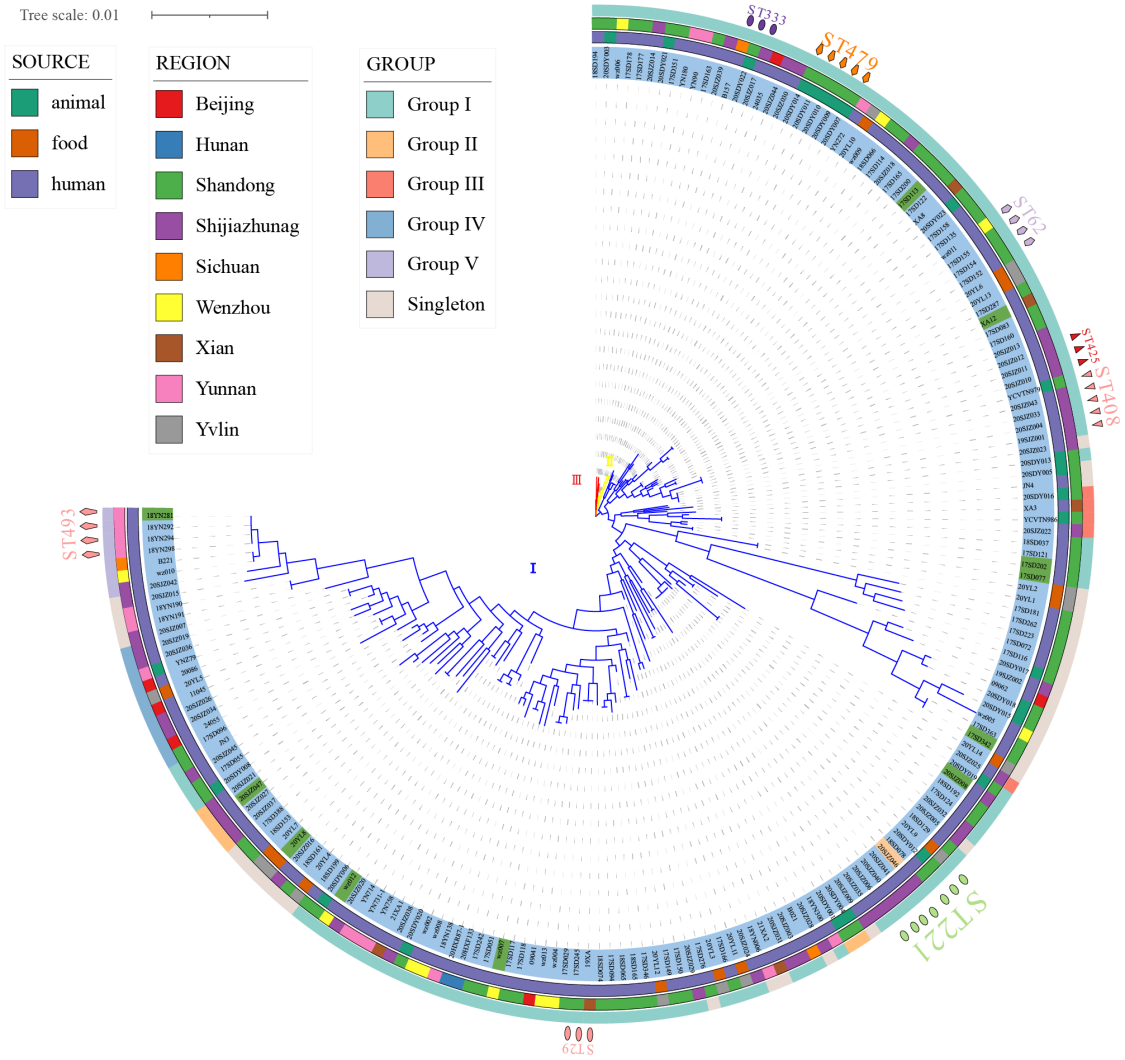
Phylogenetic maximum likelihood tree based on MLST identified in 186 strains of *Clostridium perfringens* (bootstrap test = 1,000 replicates) by fasttree. Three distinct branches are shown through the phylogenetic tree (blue, cluster I; yellow, cluster II; red, cluster III). The figure shows the population structure of the strains with *toxinotype* next to the ML tree (blue, type A; green, type F; and orange, type D), followed by the region, source, and ST of isolation as in the legend. The phylogenetic tree was visualized using iTOL.

A total of 186 *C. perfringens* strains were recovered and cultivated in our lab and characterized using 16S rRNA molecular technique. These isolates were obtained from people, including patients and healthy people, domestic animals (sheep, calves, and pigs), and food from nine different regions across China, from 2013 to 2021 (Figure 1). There were 47 isolates from Shijiazhuang, 81 isolates from Shandong, 16 isolates from Yunnan, three isolates from Sichuan, 11 isolates from Wenzhou, 14 isolates from Yulin, six isolates from Xi’an, two isolates from Hunan, and six isolates from Beijing (Figure 1). Among these 186 strains, 139 strains were from clinical sources; 24 strains were from animal sources and 14 were from food sources. Supplementary Table S1 contains a complete list of isolates background found in this study.

### Isolation and identification of *C. perfringens*

After ethanol shock treatment, all fecal specimens were inoculated with 5% egg yolk on selective tryptose sulfite cycloserine (TSC) agar (Oxoid, Basingstoke, UK) plates and incubated at 37°C for 48 h in an anaerobic jar (Mart, NL). Following vacuuming, an anaerobic atmosphere of 80% nitrogen, 10% hydrogen, and 10% carbon dioxide was injected. *C. perfringens* colonies were identified based on biochemical tests, colony and cell morphology, and other characteristics, and the 16S rRNA gene was used to identify the bacteria (short Gram-positive bacilli, dual hemolysis, and gelatinase and pectinase-producing bacteria) (Kikuchi et al., 2013).

### DNA extraction

All isolates used in this study were maintained on BHI plates with 5% sheep agar under anaerobic conditions. The genomic DNA of these isolates was extracted using a bacterial genomic DNA extraction kit (Qiagen, Germany) according to the manufacturer’s instructions. DNA was then dissolved in 100 μL of TE (10mMTris-HCl1mM EDTA PH = 8.0) and stored at −20°C until use.

### MLST and phylogenetic analysis

The eight gene loci (*colA, groEL, sodA, plc, gyrB, sigK, pgk*, and *nadA*) listed in the public *C. perfringens* MLST database^1^ were used in this study (Table 1) (Xiao et al., 2012). PCR assays were performed in final volumes of 25 μL containing 12.5 μL of 2 × PCR Taq Mastermix (MgCl, dNTP, Taq enzyme) (Takara Bio Inc., Japan); 0.5 μL of each primer (10 mmol/L); 1.5 μL of DNA template; and double-distilled water for a final volume of 25 μL. Reactions were performed with initial denaturation at 94°C for 2 min, followed by 35 cycles at 94°C for 30 s, at 55°C for 60 s and at 72°C for 60 s, and a final extension at 72°C for 8 min. Then, the PCR products were purified using a commercial kit (Omega Bio-Tek, United States) and were sent for Sanger nucleotide sequencing in both directions (Beijing DIA-UP BIOTECH Co., Ltd., China).

**Table 1:** The primer sequences of *Clostridium perfringens* housekeeping genes.

Gene	Sequence (5′-3′)	Annealing temp (°C)
*gyrB* (735 bp)	F:ATTGTTGATAACAGTATTGATGAAGC R:ATTTCCTAATTTAGTTTTAGTTTGCC	55°C
*sigK* (702 bp)	F:CAATACTTATTAGAATTAGTTGGTAG R:CTAGATACATATGATCTTGATATACC	55°C
*sod* (364 bp)	F:CAAAAAAAGTCCATTAATGTATCCAG R:TTATCTATTGTTATAATATTCTTCAC	55°C
*groEL* (685 bp)	F:TACAAGATTTATTACCATTACTTGAG R:CATTTCTTTTTCTGGAATATCTGC	55°C
*pgk* (681 bp)	F:GACTTTAACGTTCCATTAAAAGATGG R:CTAATCCCATGAATCCTTCAGCGATG	55°C
*nadA* (689 bp)	F:ATTAGCACATTATTATCAAATTCCTG R:TTATATGCCTTTAATCTTAAATCCTC	55°C
*colA* (670 bp)	F:ATTAGAAAGTTTATGTACAATAGGTG R:AAGACATTCTATTATTTCTATCGTAAGC	55°C
*plc* (630 bp)	F:AGGAACTCATGATTGTAACTC R:GGATCATTACCCTCTGATACATCGTG	55°C

**Figure 2:**
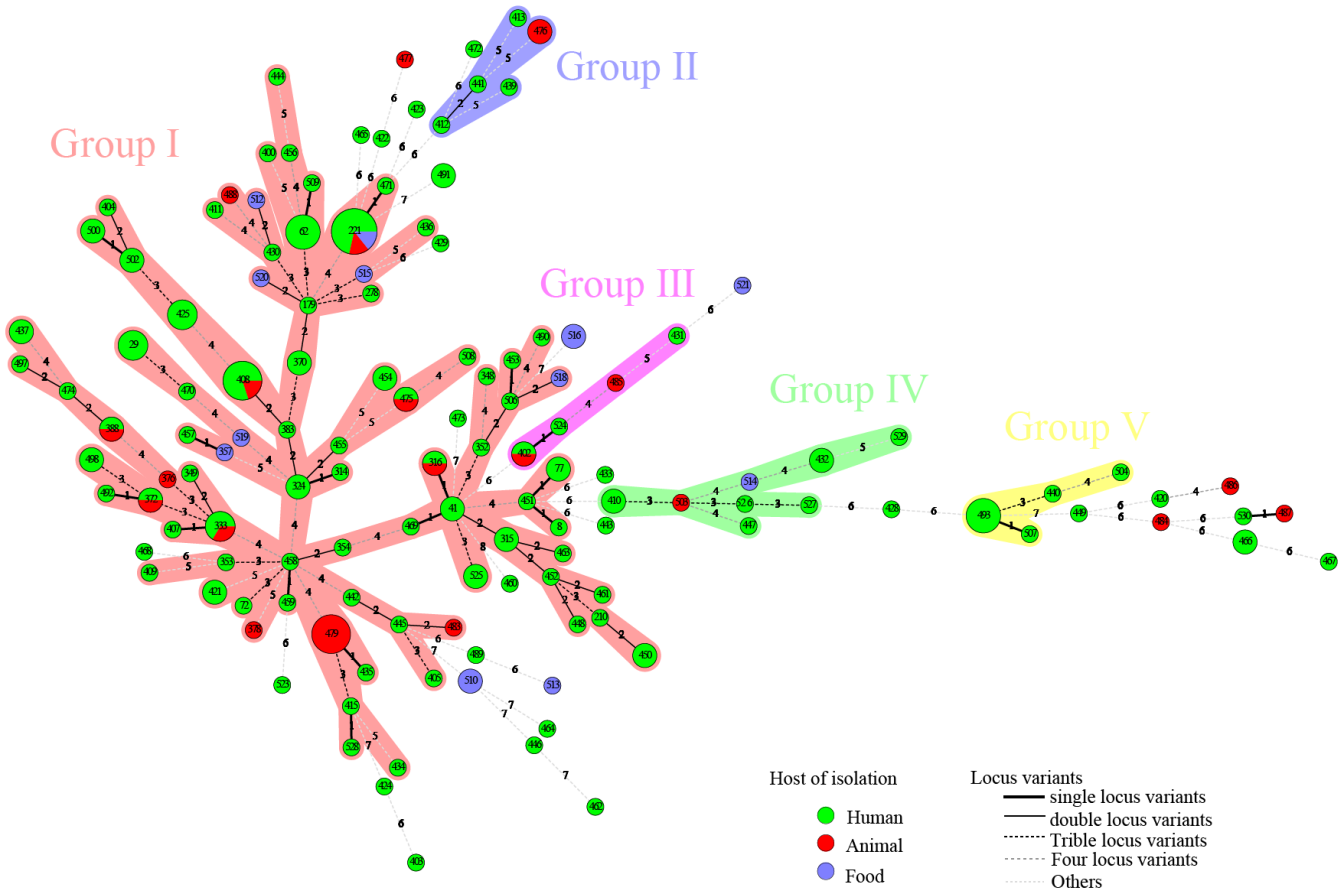
A total of 186 strains of *C. perfringens* from different sources were analyzed by constructing an MLST-minimal spanning tree. The minimum-spanning tree was constructed using the Bionumerics software (Bionumerics, version 5.1). The shaded section represents five clusters. The clusters were assigned for at least three types within five changes of neighbor distance. The area of the circle represents the number of strains; different colors represent different hosts; and the number on the branch represents the difference compared to housekeeping genes.

To determine the sequence type (ST) of all the tested 186 strains of *C. perfringens*, DNA sequences were submitted to a public *C. perfringens* MLST database (see text footnote 1) (Jolley et al., 2018). New alleles and STs were assigned in the *C. perfringens* MLST database. The minimum-spanning tree was constructed using BioNumerics software version 5.1. The tree in Figure 1 is based on nucleotide sequences, while the tree in Figure 2 is based on the allele matrix.

835 isolates were retrieved from public *C. perfringens* MLST database (see text footnote 1). Global *C. perfringens* MLST analysis was performed by online analysis on PubMLST.^2^

### Toxinotype detection

Using previously described multiplex PCR assays, the isolates were examined for the presence of the *cpa*, *cpb*, *etx*, *iap*, *cpb2*, *cpe*, and *netB* genes (Yoo et al., 1997; Bailey et al., 2013; Hu et al., 2018; Rood et al., 2018). In this investigation, the multiplex PCR was carried out using the reference strains *C. perfringens* NCTC 528 (*cpa*), NCTC 3180 (*cpb*), NCTC 4989 (*cpb*, *cpb2*), NCTC 8346 (*etx*), NCTC 8084 (*iap*, *cpe*), and NetB-encoding plasmid (pNetB) as positive controls.

### Sequencing and whole-genome sequences analysis

Genomic DNA was extracted and purified using the Wizard genomic DNA purification kit (Promega, United States). Whole-genome sequencing was carried out by Illumina HiSeq X 10 sequencing platform. Removing adapter sequences and low-quality sequences was performed using Trimmomatic version 0.39. Assembly was performed with SPAdes v3.11.1.

### cgMLST and evolutionary relationship analysis

A *C. perfringens* cgMLST scheme established by Abdel-Glil et al. (2021b) comprising 1,431 target loci based on genomic sequences was used, and neighbor-joining was used to create dendrograms based on cgMLST information via the PubMLST website (see text footnote 1).

An alignment of SNPs in the core genome using ATCC 13124 as a reference was constructed using Snippy (version 4.6) and the maximum likelihood tree was constructed using fasttree (version 2.1.10).

### Antimicrobial susceptibility test

The *E*-test method was applied for antimicrobial susceptibility analysis (Liofilchem s. r. l., Roseto degli Abruzzi, Italy) of the *C. perfringens* isolates. The following eight antimicrobials were tested: clindamycin, chloramphenicol, vancomycin, cefoxitin, meropenem, metronidazole, penicillin, and moxifloxacin. Penicillin and Clindamycin are the antibiotics of first choice for treating infections caused by *C. perfringens*. Other drugs are also common drugs in drug resistance studies of *C. perfringens* with powerful bactericidal action against gram-positive bacteria or anaerobes.

Isolates were suspended in Brucella broth to achieve a turbidity of 1.0 McFarland, and then inoculated onto Brucella agar supplemented with 5% sheep blood, 1 mg/L Vitamin K1, and 5 mg/L Hemin Chloride. Plates were incubated at 37°C for 18–24 h in anaerobic conditions. *C. perfringens* ATCC 13124 was used as a quality control. MDR was characterized as resistance to at least one agent in three or more antimicrobial classes (Magiorakos et al., 2012).

The interpretation criteria for Gram-positive anaerobic bacteria are available from the Clinical and Laboratory Standards Institute (CLSI) (Ferraro, 2001) and the European Committee on Antimicrobial Susceptibility Testing (EUCAST) (Giske et al., 2022). MIC breakpoints for clindamycin (≥8 μg/mL), chloramphenicol (≥32 μg/mL), cefoxitin (≥64 μg/mL), meropenem (≥16 μg/mL), metronidazole (≥32 μg/mL), penicillin (≥2 μg/mL), moxifloxacin (≥8 μg/mL) (Ferraro, 2001), and vancomycin (>2 μg/mL) (Giske et al., 2022) were used to read the breakpoints.

### Antibiotic resistant gene profiles

Antimicrobial resistance genes were screened using the ABRicate pipeline^3^ on the Comprehensive Antibiotic Resistance Database (CARD).^4^ Query genes with an identity higher than 80% and a coverage higher than 50% were considered potential antimicrobial resistance genes.

## Results

### Evolution and phylogenetic analysis of *C. perfringens* isolates in China by MLST

MLST analysis revealed that 186 *C. perfringens* isolates exhibited 135 sequence types (STs) with a high degree of genetic diversity, including 93 new STs. In the segments of *colA* (Kikuchi et al., 2013), *groEL* (Álvarez-Pérez et al., 2017), *sodA* (Jolley et al., 2018), *plc* (Rood et al., 2018), *gyrB* (Abdel-Glil et al., 2021b), *sigK* (Bendary et al., 2022), *pgk* (Xu et al., 2021), and *nadA* (Jolley et al., 2018), up to 129 new alleles were identified, resulting in new genotypes. Among the 135 STs, the most frequent ST was ST221 (7/186, 3.74%), followed by ST408 (5/186, 2.67%), ST479 (5/186, 2.67%), ST62 (4/186, 2.14%), and ST493 (4/186, 2.14%). There were 102 STs containing only one isolate. The 186 strains were from humans (*n* = 147), animals (*n* = 25) and food (*n* = 14) (Figure 2). The human strains comprised 110 STs, with ST221 (5/147), ST62 (4/147), ST408 (4/147), and ST493 (4/147) being the predominant STs. The animal strains contained 19 STs, with ST479 (5/25) and ST476 (2/25) being the predominant STs (Figure 2). Supplementary Table S1 contains a complete list of STs found in this study.

Of the 186 total examined isolates, 174 (93.55%) belonged to type A; 11 isolates (5.91%) were classified to type F; one isolate (0.54%) was classified to type D (Figure 1); and none of the investigated isolates were types B, C, and/or E. Clustering of the 186 genomes revealed that 91.94% (*n* = 171) belonged to cluster I, followed by cluster II (*n* = 10) and III (*n* = 5) (Figure 1). Cluster I, the most abundant subgroup, contained all predominant STs (ST221, ST408, ST479, ST62, and ST493), all *toxinotype*s (A, F, D), and all sources and all regions (Figure 1). As described, cluster I was associated with isolates from humans, food, and animals; and this cluster had a broad ecological distribution (Figure 1). *C. perfringens* type F and type D strains were also concentrated in cluster I (Figure 1). Cluster II contained ST333 (*n* = 3) as the predominant type and also included ST372 (*n* = 2) and five unique STs (Figure 1). Cluster III contained five isolates with different STs (Figure 1). Two animal isolates and eight human isolates were included in cluster II, while one animal isolate and four human isolates were included in cluster III (Figure 1). Strains in cluster II and cluster III were all type A.

There were no significant host, geographic distribution, or *toxinotype* connections among the strains involved (Figure 1). In the phylogenetic maximum likelihood tree, there were several STs, including isolates with different regions, hosts, or different *toxinotype*s within the same ST such as ST221, ST408, ST333, ST372, ST316, ST388, ST402, and ST475, indicating that isolates may have potentially spread across different hosts or regions (Figure 1). For example, ST221 contained seven strains from clinical diarrhea patients [Shijiazhuang (*n* = 4), Shandong (*n* = 1)], sheep [Shandong (*n* = 1)] and food [Yulin (*n* = 1)] (Figure 1). ST408 consisted of five strains originating from AAD patients [Shijiazhuang (*n* = 4)] and a calf [Shandong (*n* = 1)] (Figure 1).

A total of five clusters (cluster I–cluster V) were found in the minimal spanning tree (Figure 2) among the studied strains isolated in this investigation, accounting for 82.80% (154/186) of the strains. Thirty-two STs were determined to be singletons, with no observed cluster relationships. Cluster I, the most prolific cluster, included 80 STs (including advantage STs ST221, ST408, ST479, ST62, ST333, and ST493), with a total of 122 strains that accounted for 65.59% (122/186) of all detected strains. Cluster II contained four clinical origin strains (ST412, ST413, ST439, and ST441) and two animal origin strains (ST476), accounting for 3.23% (6/186) of the examined strains. Cluster III contained three clinical origin strains (ST402, ST431, and ST524) and two animal origin strains (ST402 and ST485), accounting for 2.69% (5/186) of the examined strains. Cluster IV contained eight human origin strains (ST410, ST432, ST447, ST526, ST527, and ST529), one animal origin strain (ST503), and one food origin strain (ST514), accounting for 5.38% (10/186) of the examined strains. Cluster V contained seven human origin strains (ST440, ST493, ST504, and ST507), accounting for 3.76% (7/186) of the examined strains; these showed genetic distance (≥6 allele differences) with other isolates in the minimum-spanning tree (Figure 2) and significant genetic distance (≥7 allele differences) with other clusters (Figure 2). Half of the food origin strains were dispersed as singletons, while most strains with animal origin clustered with strains of human origin, indicating the character of the zoonotic food-borne pathogen *C. perfringens*.

**Figure 3:**
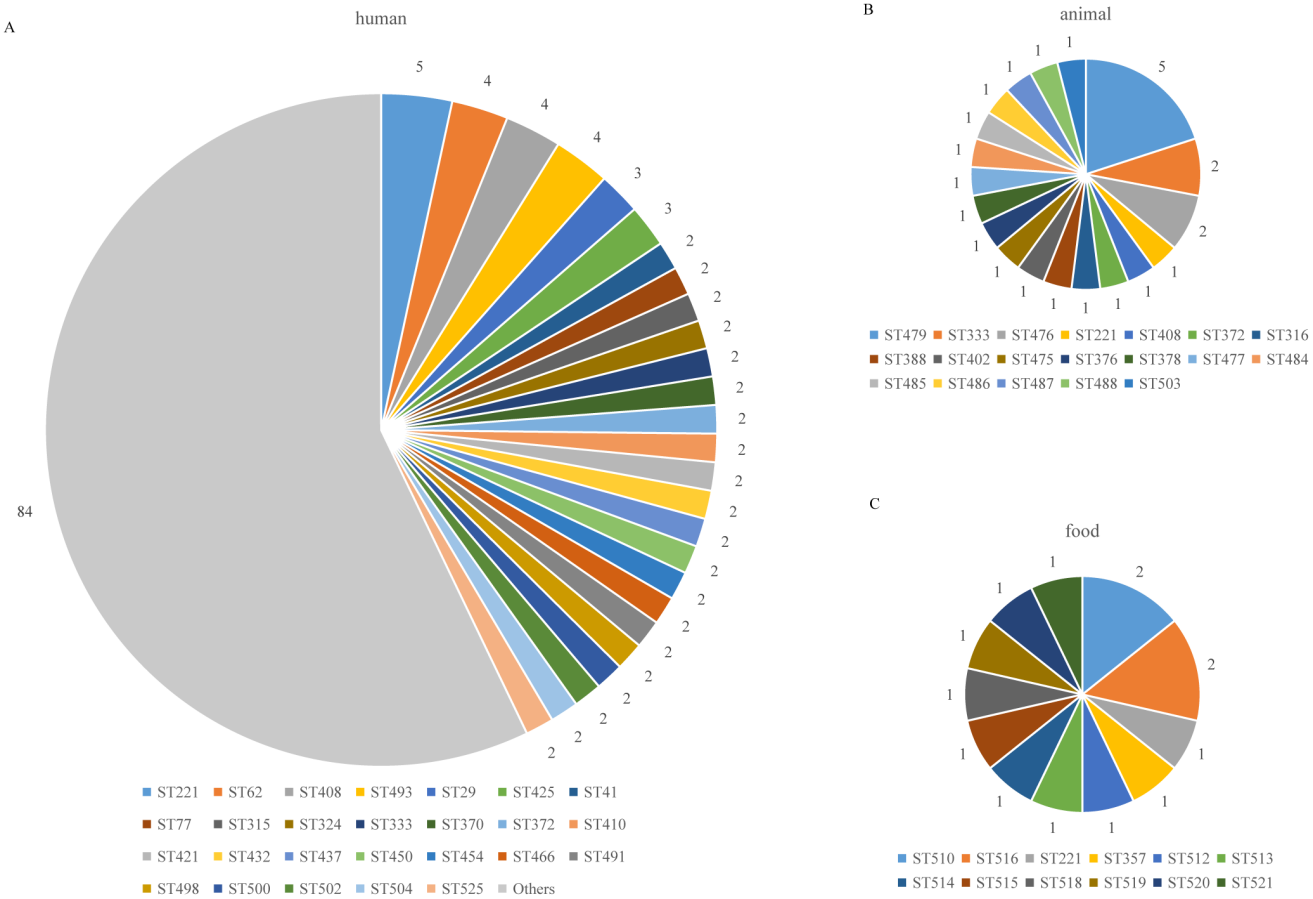
Distribution of *C. perfringens* ST types from different hosts. The “Others” include ST types with only 1 isolate; the number of other STs was 84: **(A)** human **(B)** animal **(C)** food.

Interestingly, the distribution of isolates from multiple sources in the minimum-spanning tree (Figure 2) and in the phylogenetic tree (Figure 1) was not consistent. Due to different mathematical calculation models (the minimum-spanning tree was based on a matrix, while the phylogenetic tree was based on nucleic acid sequences), some strains belonging to similar branches in the phylogenetic tree did not form the same clusters in the minimum-spanning tree. For example, some isolates in cluster I were assigned to different clusters of the minimum-spanning tree (Figures 2, 3).

### Global *C. perfringens* MLST analysis

**Figure 4:**
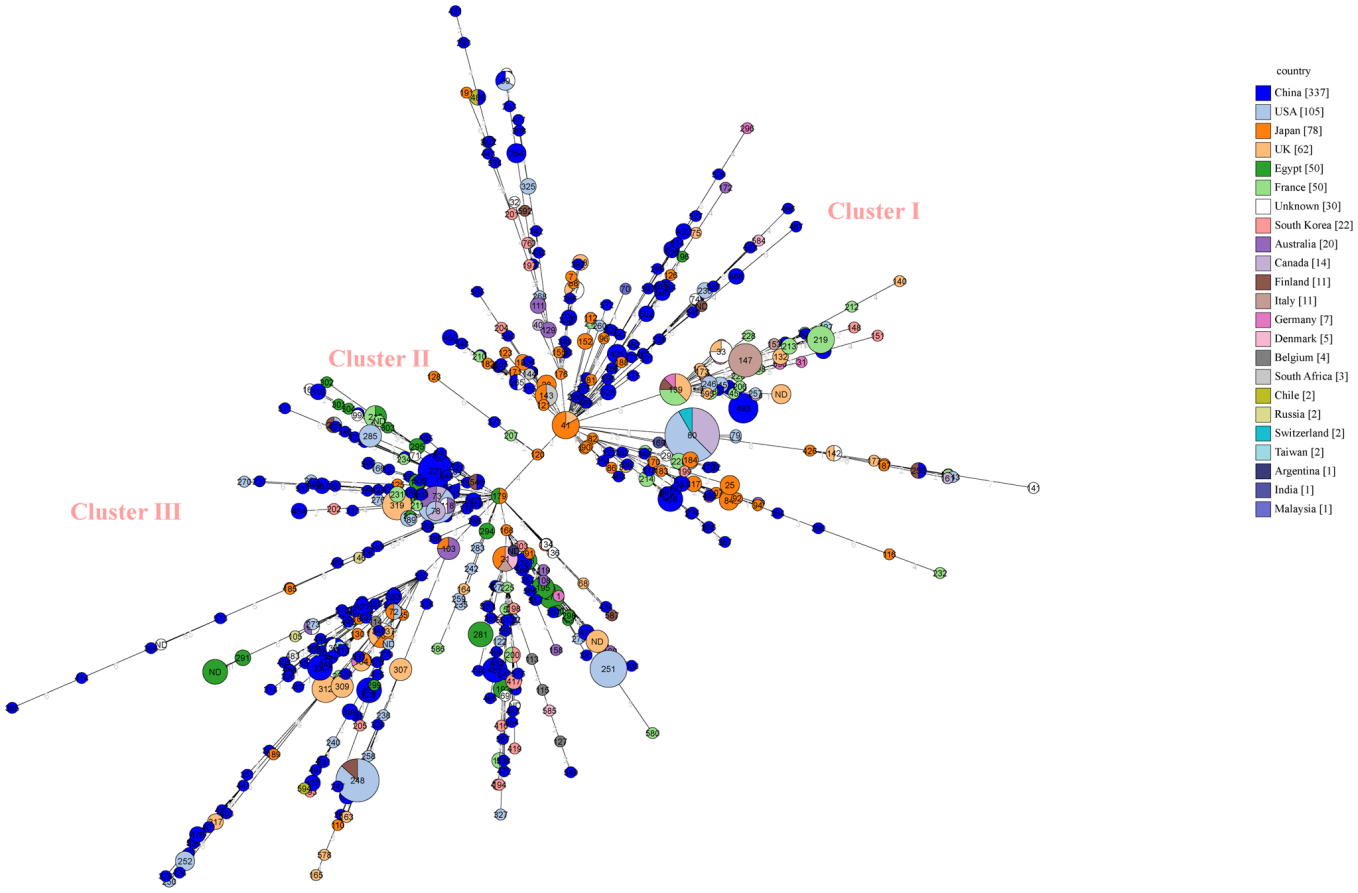
Minimum spanning tree (MST) of MLST for global *C. perfringens* as generated within PubMLST using GrapeTree. MST representing evolutionary relationships of *C. perfringens* sequence types (STs) between Chinese isolates and other 21 countries isolates. Isolates from one country were marked by one color. The size of the circle is corresponding to the number of isolates within that genotype. Numbers inside circles are STs and numbers over connecting lines are locus variant levels.

We performed MLST analysis on 835 strains isolated from different sources (clinical, animal, food and environment) (Figure 4). A total of 592 STs were identified (Figure 4). The global strains can be divided into three distinct clusters, and Chinese strains from database (*n* = 337), scattered among the three clusters (Figure 4). Japanese strains are mostly clustered in clusterI (Figure 4). The majority single ST types correlated to one isolate from one country (Figure 4). The main predominant types globally were ST80 (*n* = 24), ST248 (*n* = 15), ST251 (*n* = 11), ST73 (*n* = 11), ST221 (*n* = 10) (Figure 4). Among them, ST80, ST248, and ST73 are found in multiple nations (Figure 4). The molecular type ST80 strains came from the United States (*n* = 13), Canada (*n* = 9), and Switzerland (*n* = 2), the ST248 strains came from the United States (*n* = 13) and Finland (*n* = 2), and the ST73 strains came from the United States (*n* = 7), Australia (*n* = 3), and Canada (*n* = 1).

### Cgmlst and cgSNP evolutionary relationship analysis of the same STs

Figure 3 shows the genetic relatedness of strains in the same STs on the cgMLST scheme from PubMLST based on the presence of the genes and sequences of 1,431 highly conserved core genes.

**Figure 5:**
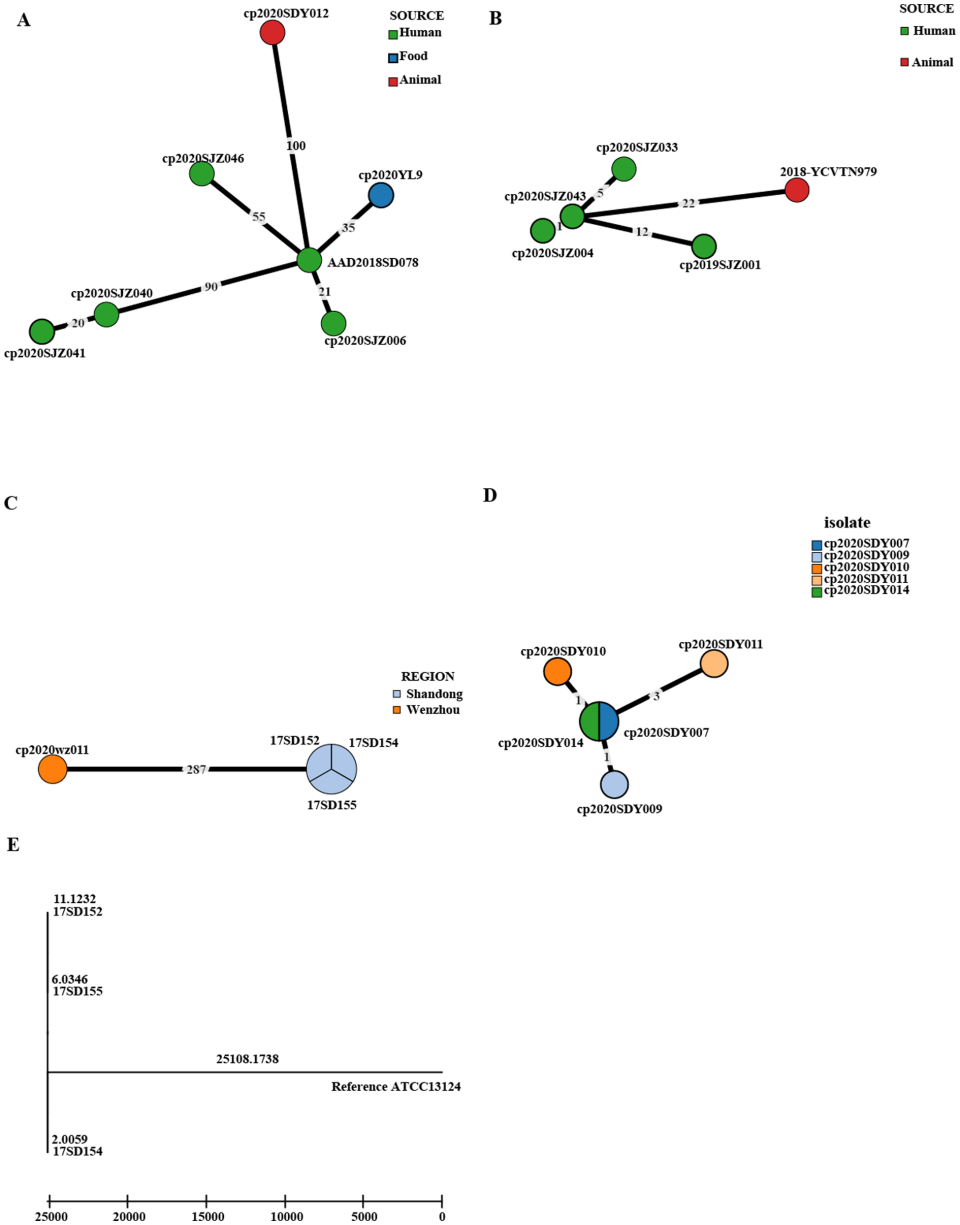
Phylogenetic tree of cgMLST and core SNP typing. **(A–D)** GrapeTree minimum-spanning tree of strains in the same STs based on the PubMLST cgMLST. The numbers on the branches represent the differences in alleles between neighboring nodes. **(A)** ST221, **(B)** ST408, **(C)** ST62, and **(D)** ST479. **(E)** A maximum likelihood (ML) phylogenetic tree of ST62 based on SNPs constructed by snippy and fasttree version 2.1.10 using ATCC 13124 as a reference.

Isolates with the same ST221 were divided into seven cgMLST types, comprising five from clinical human isolates, one from food, and one from animals (Figure 5A). The seven genomes showed ≤100 allele differences (range 20–100). Furthermore, the food isolate cp2020YL9 (ST221) and sheep isolate cp2020SDY012 (ST221) showed 35 and 100 allele differences, respectively, compared to the human cp2018SD078 (ST221) isolate, indicating that these isolates with identical ST were genetically unrelated (Figure 5A).

In addition, within the ST408 complex, fetal cattle isolate 2018-YCVTN979 showed 22 allele differences compared to human isolate cp2020SJZ043, indicating that these isolates with identical cgMLST type were genetically heterogeneous (Figure 5B). However, the three human clinical isolates cp2020SJZ004, cp2020SJZ043, and cp2020SJZ033 showed high genetic relatedness with ≤5 allele differences.

Moreover, the three AAD strains with the same ST 62 isolated from the same hospital at Shandong in 2017 (17SD152, 17SD154 and 17SD155) were also classified to the same cgMLST type showing no allele differences (Figure 5C), strongly suggesting that they had arisen from a single source or were associated with the same transmission event. To confirm our hypothesis, cgSNP analysis was further performed for these three isolates (Figure 5E). The size of the core genome for *C. perfringens* isolates included in this study was 22,493 bp, and the average size of the *C. perfringens* genome for isolates sequenced in this study was 3.26 Mb. A phylogenetic tree was produced using the 6,914 bp core genome SNP alignment. Within 6,914 variant sites, pairwise SNP diversity ranged from 20 to 22 SNPs for the three genomes (Supplementary Table S1), illustrating that these three isolates had clear genetic differences. Notably, there was another strain (cp2020wz011) in the ST 62 complex that displayed 287 allele differences with the above three AAD strains (Figure 5C).

Furthermore, within the ST 479 complex, five isolates from sheep in Shandong were divided into four cgMLST types, including cp2020SDY007 and cp2020SDY014 that belonged to the same cgMLST type (Figure 5D). The five genomes showed ≤3 allele differences (range 0–3) below the cutoff threshold of seven allele differences.

### Antibiotic-resistance profiles

**Figure 6:**
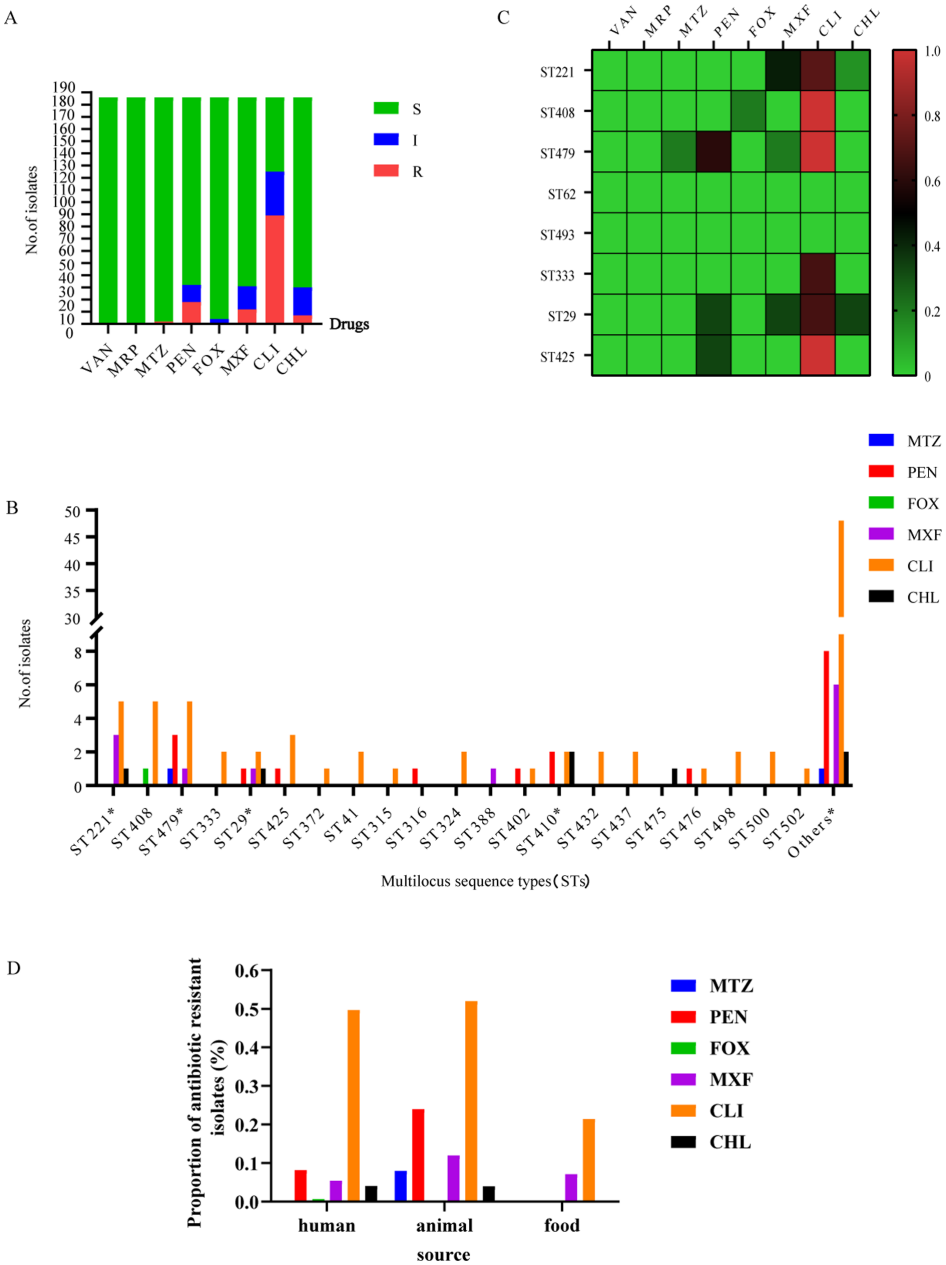
Antibiotic-resistance profiles of 186 *C. perfringens* strains. **(A)** Isolates sensitive, intermediate, or resistant to antimicrobial agents for 186 *C. perfringens* strains. S, sensitive; I, intermediate; and R, resistant. **(B)** Number of drug-resistant strains of different ST types of the 186 *C. perfringens* isolates.*, MDR strain. The “Others” group include resistance to antibiotics of ST types with only 1 strain; the number of other STs was 102. metronidazole (MTZ), penicillin (PEN), cefoxitin (FOX), moxifloxacin (MXF), clindamycin (CLI), chloramphenicol (CHL). **(C)** The heatmap correlating ST with antibiotic resistance. The percentages of resistance to antimicrobials are color-coded on the right side of the figure. Colors represent the proportion of ST-type resistance. Red indicates high resistance, brown indicates medium resistance and green indicates low resistance. The data analyzed here are from samples ≥3. **(D)** The percentages of resistant strains presented as the three different groups -human, animal and food.

Of the 186 strains tested, 96 were resistant to at least one antibiotic. All of the *C. perfringens* isolates showed 100% susceptibility to vancomycin and meropenem. The rates of resistance to metronidazole, penicillin, cefoxitin, moxifloxacin, and chloramphenicol were low (metronidazole: 1.08%; penicillin: 9.68%; cefoxitin: 0.54%; moxifloxacin: 6.45%; and chloramphenicol: 3.76%) (Figure 6A). However, these isolates displayed much higher rates of antibiotic resistance to clindamycin (47.85%) (Figure 6A).

These antibiotics (metronidazole, cefoxitin, moxifloxacin and chloramphenicol) with lower resistance rates, ST29 was the major molecular type of antibiotic-resistant *C. perfringens* (Figure 6B). Furthermore, there was an MDR strain (AAD2019XAzhou) in ST29 (Figure 6B). Moreover, genotypes ST408 and ST479 were the predominant types among clindamycin-resistant isolates, followed by ST221 and ST425 (Figure 6B). Overall, ST479, ST408, ST29, and ST410 were the most commonly identified genotypes that correlated with a high proportion of antibiotic resistance (Figure 6C). Moreover, the strains in cluster V were sensitive to all of the above antibiotics (Supplementary Tables S1, S2).

For isolates of human origin, all were susceptible to vancomycin, meropenem, and metronidazole. The rates of cefoxitin (0.68%), chloramphenicol (4.08%), moxifloxacin (5.44%), and penicillin (8.16%) resistance were low. The resistance rate to clindamycin (49.66%) was much higher (Figure 6D). For isolates of animal origin, all were susceptible to vancomycin, cefoxitin, and meropenem. The antibiotics to which the tested isolates were resistant were clindamycin (52%), penicillin (24%), moxifloxacin (12%), metronidazole (8%), and chloramphenicol (4%) (Figure 6D). For isolates of food origin, all isolates were susceptible to vancomycin, meropenem, metronidazole, penicillin, cefoxitin, and chloramphenicol (Figure 6D). The resistance rates of moxifloxacin and clindamycin were 7.14 and 21.43%, respectively (Supplementary Table S1).

The antimicrobial susceptibility testing showed that the multidrug resistance of isolates was low in this study (Figure 6B), with a proportion of multidrug-resistant isolates of 3.23% (6/186). There were two MDR strains in ST410, one MDR strain in ST29, one MDR strain in ST221, one MDR strain in ST443, and one MDR strain in ST479 (Figure 6B) isolated from sheep (*n* = 1) and humans (*n* = 5). The MDR proportion of human isolates was 3.40% (5/147). The MDR proportion of animal isolates was 4% (1/25). There were no multidrug-resistant strains of food origin in our study. Supplementary Table S1 contains a complete list of antibiotic-resistance profiles found in the study isolates.

### Antibiotic resistant gene profiles

The carriage of resistance genes in *C. perfringens* is shown in Table 2, with specific information in Supplementary Table S3. The resistance genes with high carriage rates were as following: 100% *C. perfringens* carried the peptide resistance gene *Clostridium_perfringens_mprF.* The carriage rates of tetracycline resistance genes *tetA(P)*, *tetB(P)*, and *tet44* were 97.19, 79.21, and 20.79%, respectively. The carriage rates of macrolide/lincosamide/streptomycin (MLS) resistance genes *ErmQ*, *LnuP*, and *ErmB* were 52.25, 33.71, and 8.99%, respectively. Antibiotics referred to as aminoglycosides, including *ANT(6)-Ib* and *AAC(6′)-Ie-APH(2″)-Ia*, had carriage rates of 21.35 and 20.79%, respectively.

**Table 2:** The rates of antibiotic resistance genes.

The rates of antibiotic resistance genes		
Gene	Number	(%)
*Clostridium_perfringens_mprF*	178	100.00%
*tetA(P)*	173	97.19%
*tetB(P)*	141	79.21%
*ErmQ*	93	52.25%
*LnuP*	60	33.71%
*AAC(6′)-Ie-APH(2″)-Ia*	38	21.35%
*ANT(6)-Ib*	37	20.79%
*tet44*	37	20.79%
*ErmB*	16	8.99%
*ErmA*	4	2.25%
*fexA*	4	2.25%
*optrA*	4	2.25%
*mphB*	3	1.69%
*ErmT*	1	0.56%
*dfrF*	1	0.56%
*lnuD*	1	0.56%
Total	178	100%

## Discussion

To the best of our knowledge, this is the first relatively comprehensive cross-sectional study concerning the molecular characterization of *C. perfringens* isolates in China. In our study, samples from patients, healthy humans, animals, and food were subjected to MLST analysis, *toxinotype* analysis, cgMLST analysis, and antibiotic susceptibility testing.

The MLST analysis of Chinese strains indicated a wide genetic diversity of *C. perfringens*, as previously reported (Verma et al., 2020). In our study, the predominant STs from human isolates (ST221, ST62, ST408) were significantly distinct from the predominant STs (ST479) from animal isolates, a result that was consistent with previous research on global *C. perfringens* strains, indicating a diverse distribution of *C. perfringens* among multiple sources (Verma et al., 2020; Wang et al., 2021). In global surveys, a clonal complex (CC) represented by three STs (ST98, ST41, and ST110) predominantly representing type F (18/20 strains) was mostly associated with human illnesses (Verma et al., 2020). Among the predominant STs, ST 54 was associated with enteritis cases in foals and dogs, and ST 58 was associated with necrotic enteritis in poultry (Verma et al., 2020). In our study, most type F strains (10/11 strains) were associated with human illnesses, different from the results of previous studies.

Global MLST analysis indicates a wide genetic diversity of *C. perfringens*. 835 isolates represented 563 STs. Among three predominant five STs, ST 80 represented maximum number of strains and belonged to three regions (United States, Canada and Switzerland). The same ST type can occur in geographically distant countries. In the minimum spanning tree, the distribution of strains of Japanese origin is relatively concentrated. It is probably due to the fact that they are all strains of clinical origin.

In terms of strains of human origin, the isolates recovered from human diarrhea cases (*n* = 111) were found to be of type A (*n* = 102), type F (*n* = 8), and type D (*n* = 1). Type A and type F were the major *toxinotype*s in the isolates, basically in accordance with earlier reports (Aung et al., 2021; Wang et al., 2021). Interestingly, there was one isolate of type D that carried *cpe* and/or *cpb2* genes, indicating that it had the potential to produce the CPE toxin (Kiu and Hall, 2018). ST221 was the dominant genotype of *C. perfringens* among the isolates, with significant genetic diversity. Previous results revealed that ST41 and ST77 were associated with human illnesses (Verma et al., 2020; Wang et al., 2021). Consistent with these findings, we observed that the strains ST41 (2/2, type A) and ST77 (2/2, type F) were isolated from patients with diarrhea. The resistance rates of *C. perfringens* depend on the use of antibiotics in different regions. For example, some studies in Hungary, Slovenia, and northern Taiwan showed that cefoxitin, meropenem, and metronidazole were successful against *C. perfringens*. Only a minority were penicillin (2.6%) or clindamycin (3.8%) resistant (Sarvari and Schoblocher, 2020). However, in our study, we found high resistance (49.66%) to clindamycin in human origin samples in China. Similarly penicillin-resistant (10.2%) and penicillin-insensitive strains (18.2%) were much more frequent in this study than in previous studies. Moreover, recent data from Italy showed that clindamycin resistance was >20% for *Clostridium* spp. (Di Bella et al., 2022). In fact, penicillin and clindamycin have long been considered excellent drugs for *Clostridial* infections (Satyaprakash et al., 2010). In particular, penicillin is currently the drug of first choice for *C. perfringens* infections. Thus, our findings suggest that the two antibiotics should be used with caution as empirical therapies in China.

Among the strains of animal origin, all were found to be type A in this study. A relatively high prevalence of MDR (73.4%) was found among the isolates from different animals in a previous study (Xiaoting et al., 2021). The MDR profile was resistant against tetracycline (84%), erythromycin (72%), sulfamethoxazole (97.3%), trimethoprim (96.3%), and streptomycin (84.4%) (Xiaoting et al., 2021). Interestingly, the prevalence of MDR *C. perfringens* strains was rather low in this study (4%, 1/25 animals), possibly due to different choices of tested antimicrobials. The small scale of animal samples or low use of antibiotics in selected farms is also potential cause. Furthermore, the environment in which *C. perfringens* lives (e.g., the gut) may play a significant role in drug resistance. Therefore, in the future, close monitoring is necessary for a more thorough investigation of antibiotic resistance of *C. perfringens* in China. Rational antibiotic use may play an important role in reducing the drug resistance of *C. perfringens*.

*C. perfringens* is an important microorganism as a foodborne pathogen. Among the *C. perfringens* of food origin, in a previous study in Egypt, most of the food origin isolates (74%) (beef, chicken meat, and raw milk) exhibited MDR patterns. Strains isolated from raw camel milk in Isiolo County, Kenya, had high resistance to chloramphenicol (42.37%), kanamycin (40.68%), tetracycline (37.29%), and gentamycin (35.59%) (Aliwa et al., 2019). Another study in Korea reported that resistance to chloramphenicol (26/38, 68.4%) and metronidazole (13/38, 34.2%) was observed in *C. perfringens* from retail meats (Jang et al., 2020). Surprisingly, in our study, all of the isolates from retail meat products were susceptible to chloramphenicol and metronidazole, and none of the isolates showed an MDR pattern. Given that most of the samples in this study were of human origin, the selected antimicrobials were biased toward clinical use, a feature that was inconsistent with other studies using isolates of animal or food origin.

Antimicrobial resistance gene profiling among the recovered isolates revealed a higher presence of the *mprF* gene (100%) followed by *tetA(P)* (97.19%), *tetB(P)* (79.21%), *ermQ* (52.25%), and *lnuP* (33.71%) (Supplementary Table S3). These are common drug resistance genes in *C. perfringens*. All *C. perfringens* genomes had the *mprF* gene, which is known to impart resistance via altering the surface molecules of AMPs and is highly conserved in *C. perfringens* (Kiu et al., 2017). Since the 1980s, it has been known that the gene *tetA(P)* and *tetB(P)* are most frequently encoded in tetracycline efflux proteins of *C. perfringens* (Lyras and Rood, 1996). *TetA(P)* was found in 75% of the 56 strains in the prior genome investigation, which is more common than *tetB(P)* (42%) (Kiu et al., 2017). The high resistance of tetracycline (84%) was recorded out of the 50 animal origin isolates in previous study. In another study, clinical isolates (49%) as well as isolates from chickens (73%) and soil (63%) and food (42%) also were found to be tetracycline-resistant (Bendary et al., 2022). *C. perfringens* isolates from different sources varied in their tetracycline resistance levels and the frequency of the tetracycline-resistance genes *tetA(P)* and *tetB(P)* (Park et al., 2010). In this study, *ErmQ* was found to be the predominant macrolide-lincosamide-streptogramin (MLS) resistance gene of *C. perfringens* (Berryman et al., 1994). Also, *lnuP* gene conferring lincosamide resistance has been discovered in *C. perfringens* (Lyras et al., 2009). The isolates in this investigation were reported to be 52% resistant to the MLS drug clindamycin.

The three isolates (17SD152, 17SD154, and 17SD155) with the same ST62 isolated from the same hospital were assigned to the same cgMLST type (with no allele difference). Their visit times were close, no more than half a month apart. The three patients were 6 months old, 34 years old, and 62 years old. The three isolates collected at a single Shandong hospital inferred a potential transmission chain. The five isolates within the ST479 complex (cp2020SDY007, cp2020SDY009, cp2020SDY010, cp2020SDY011, and cp2020SDY014) of sheep origin in Shandong were divided into four cgMLST types with allele differences range 0–3, including cp2020SDY007 and cp2020SDY014 that belonged to the same cgMLST type (Figure 5D). Thus, we suspect that transmission of these *C. perfringens* strains in single farm took place. Genetic relatedness of strains isolated from three AAD patients from the same hospital and five sheep from the same farm in Shandong indicates that these isolates are originated from a single source or associated with the same transmission event and inter-host transfer of *C. perfringens*.

In our study, sequence type complex 221 was further divided into different cgMLST profiles. The STc221 strains across different provinces and hosts showed large allelic differences, indicating independent development and distribution. Meanwhile, this pattern showed that cgMLST had higher discrimination than the classical MLST method. Overall, cgMLST made it easy to clarify transmission routes, target the epidemiological investigation, and delineate infection control interventions. The cgSNP based on whole-genome sequencing (WGS) has advantages in understanding the dissemination of *C. perfringens.*

In conclusion, this study provides a glance at the molecular features and genetic characters of *C. perfringens* isolates in China. Using MLST analysis of 186 *C. perfringens* strains from diverse hosts, we found distinct differences in dominant STs among humans and animals. Similar characters were reported in other studies (Verma et al., 2020). Moreover, we gained insight into the antibiotic-resistance profiles in China of this significant pathogen. In our study, the percentage of MDR strains was much lower than previously reported. Interestingly, isolates of human origin were highly clindamycin-resistant and penicillin-insensitive, implying that their use as empirical therapies in China should be approached with caution. We analyzed the genetic relatedness of these specific complexes with the same STs to identify potential transmission links based on MLST, cgMLST, and cgSNP. Our results indicated that the isolates of the same ST may be genetically unrelated. cgMLST and cgSNP are more discriminant than traditional MLST and are suitable approaches for outbreak and transmission analyses. Further studies focused on *C. perfringens* of animal and food origin nationwide are needed to gain deep insights into prevention and control strategies, routine outbreak detection, and national surveillance in China.

## Data availability statement

The genomic sequencing data set was deposited in the NCBI under submission: SUB12955969 and submission SUB12688567.

## Author contributions

JZ, HZ, YW, LB and XD performed the experiments. JZ and HZ analyzed data and finished figures. JZ wrote the manuscript. YW designed the study and reviewed the manuscript. JL reviewed the manuscript. All authors read and approved the final manuscript.

## Funding

This study was supported by the National Key Research and Development Program of China (2021YFC2301000).

## Conflict of interest

The authors declare that the research was conducted in the absence of any commercial or financial relationships that could be construed as a potential conflict of interest.

## Publisher’s note

All claims expressed in this article are solely those of the authors and do not necessarily represent those of their affiliated organizations, or those of the publisher, the editors and the reviewers. Any product that may be evaluated in this article, or claim that may be made by its manufacturer, is not guaranteed or endorsed by the publisher.

## Footnotes

^1^https://pubmlst.org/organisms/clostridium-perfringens

^2^https://pubmlst.org/bigsdb?db=pubmlst_cperfringens_isolates&page=query&genomes=1

^3^https://github.com/tseemann/abricate

^4^https://card.mcmaster.ca/
